# Controllable synthesis of branched ZnO/Si nanowire arrays with hierarchical structure

**DOI:** 10.1186/1556-276X-9-328

**Published:** 2014-06-30

**Authors:** Shengli Huang, Qianqian Yang, Binbin Yu, Dingguo Li, Ruisheng Zhao, Shuping Li, Junyong Kang

**Affiliations:** 1 Fujian Provincial Key Lab of Semiconductors and Applications, Department of Physics, Xiamen University, Xiamen, Fujian 361005, People's Republic of China; 2 State Key Lab of Silicon Materials, Zhejiang University, Hangzhou 310027, People's Republic of China

**Keywords:** ZnO/Si nanowire arrays, Hierarchical structure, Chemical etching, Hydrothermal growth

## Abstract

A rational approach for creating branched ZnO/Si nanowire arrays with hierarchical structure was developed based on a combination of three simple and cost-effective synthesis pathways. The crucial procedure included growth of crystalline Si nanowire arrays as backbones by chemical etching of Si substrates, deposition of ZnO thin film as a seed layer by magnetron sputtering, and fabrication of ZnO nanowire arrays as branches by hydrothermal growth. The successful synthesis of ZnO/Si heterogeneous nanostructures was confirmed by morphologic, structural, and optical characterizations. The roles of key experimental parameters, such as the etchant solution, the substrate direction, and the seed layer on the hierarchical nanostructure formation, were systematically investigated. It was demonstrated that an etchant solution with an appropriate redox potential of the oxidant was crucial for a moderate etching speed to achieve a well-aligned Si nanowire array with solid and round surface. Meanwhile, the presence of gravity gradient was a key issue for the growth of branched ZnO nanowire arrays. The substrate should be placed vertically or facedown in contrast to the solution surface during the hydrothermal growth. Otherwise, only the condensation of the ZnO nanoparticles took place in a form of film on the substrate surface. The seed layer played another important role in the growth of ZnO nanowire arrays, as it provided nucleation sites and determined the growing direction and density of the nanowire arrays for reducing the thermodynamic barrier. The results of this study might provide insight on the synthesis of hierarchical three-dimensional nanostructure materials and offer an approach for the development of complex devices and advanced applications.

## Background

One-dimensional (1D) nanomaterials have received increasing attention in nanodevices and nanotechnology due to their unique properties, such as large surface-to-volume ratio, nanocurvature effect, and direct pathway for charge transportation [1]. Most importantly, they may be the building blocks of complex two- and three-dimensional (2D and 3D) architectures [2,3]. Among the 1D nanomaterials, Si nanowires are considered to be a promising candidate for the components of solar energy harvesting systems [4]. The advantages of Si nanowires lie in their low-energy bandgap (*E*_g_ = 1.12 eV) [4] that can absorb sunlight efficiently as well as the fundamental materials in current photovoltaic market. However, some serious troubles may be encountered in applying the Si nanowires merely in the optoelectronics and photocatalysis as photoelectrodes. First, the materials are easy to be corroded in electrolyte. Second, the Si possesses high valence band maximum energy that is thermodynamically impossible to oxidize water spontaneously [5,6]. Third, the surface-to-volume ratio may be limited for the 1D nanostructures. To address these issues, the surface of the Si nanowires can be coated by a layer of metal oxides that resists the electrolyte corrosion and also modulates the energy diagram between the Si and the electrolyte. On the other hand, the surface area can be further increased by hierarchical assembly of 1D nanostructures into 2D or 3D nanostructures. In this sense, 3D branched ZnO/Si or TiO_2_/Si nanowire arrays with hierarchical structure are the most favorite choice, as the ZnO and TiO_2_ nanowire branches not only extend the outer space above the substrate but also display stable physical and chemical properties in electrolytes [5,7-9]. In addition, the conduction and valence band-edges of ZnO and TiO_2_ just straddle H_2_O/H_2_ and OH^−^/O^2−^ redox levels and thus satisfy a mandatory requirement for spontaneous photosplitting of water [10]. In contrast with TiO_2_, ZnO is more flexible to form textured coating in different types of nanostructures by anisotropic growth [11-14]. Therefore, the branched ZnO/Si nanowire arrays with hierarchical structure have attracted more and more researchers' interest since their first successful synthesis in 2010 [9,15-20]. Want et al. fabricated the ZnO/Si nanowire arrays by a solution etching/growth method and applied them in photodetectors [15]. The specimen presented a high photodetection sensitivity with an on/off ratio larger than 250 and a peak photoresponsivity of 12.8 mA/W at 900 nm. They also used them in photoelectrochemical cells and found that the 3D nanowire heterostructures demonstrated large enhancement in photocathodic current density (an achieved value as high as 8 mA/cm^2^) and overall hydrogen evolution kinetics [16]. Kim synthesized the ZnO/Si nanowire arrays by combining nanosphere lithography and solution process [9]. The sample was used in solar cells and exhibited an enhanced photovoltaic efficiency by more than 25% and an improved short circuit current by over 45% compared to the planar solar cells. Nevertheless, all the above reports are chiefly concentrating on the specimen's performance either on photocatalysis or on optoelectronics. The basic issues, the growth mechanism and the role of key growth parameters on the hierarchical structure formation, are actually neglected. Since the function of the ZnO/Si nanowire arrays primarily depends on the composition distribution and nanostructure feature, a systematic research about the influence of different growth parameters on the hierarchical nanostructure formation is crucial to the controllable synthesis as well as the related applications.

With the above considerations, in this letter, we proposed a rational routine for creating branched ZnO/Si nanowire arrays with hierarchical structure. The specimens were synthesized through growth of crystalline Si nanowire arrays as backbones first, subsequent deposition of ZnO thin film as a seed layer on the surface of the backbones, and final hydrothermal growth of ZnO nanowire branches. The successful synthesis of ZnO/Si heterogeneous nanostructures was confirmed by the results of scanning electron microscopy (SEM), energy dispersive X-ray spectroscopy (EDS), X-ray diffraction (XRD), photoluminescence (PL), and reflectance spectra. The experimental parameters, such as the solution type, the substrate direction, and the seed layer, were systematically investigated to determine the optimum growth conditions of the ZnO/Si hierarchical nanostructures.

## Methods

### Materials and reagents

P-type, boron-doped (100) Si wafers with a resistivity of 1 to 10 Ω cm and a thickness of 450 μm were purchased from Shanghai Guangwei Electronic Materials Co. Ltd (Shanghai, China). Hydrogen peroxide (H_2_O_2_) 30%, nitric acid (HNO_3_) 65%, sulfuric acid (H_2_SO_4_) 95%, hydrochloric acid (HCl) 36%, hydrofluoric acid (HF) 40%, toluene (C_6_H_5_CH_3_), acetone (C_3_H_6_O), ethanol (C_2_H_5_OH), zinc acetate dihydrate (Zn(CH_3_COO)_2_ · 2H_2_O), and hexamethylenetetramin (C_6_H_12_N_4_) were all bought from Xilong Chemical Co. Ltd (Guangdong, China). Silver nitrate (AgNO_3_) was ordered from the First Regent Factory (Shanghai, China). Distilled water (H_2_O) with resistivity higher than 18.0 MΩ cm was purified by a hi-tech laboratory water purification system. All the solvents and chemicals used in the experiments were at least reagent grade and were used as received.

### Synthesis process

**Figure 1:**
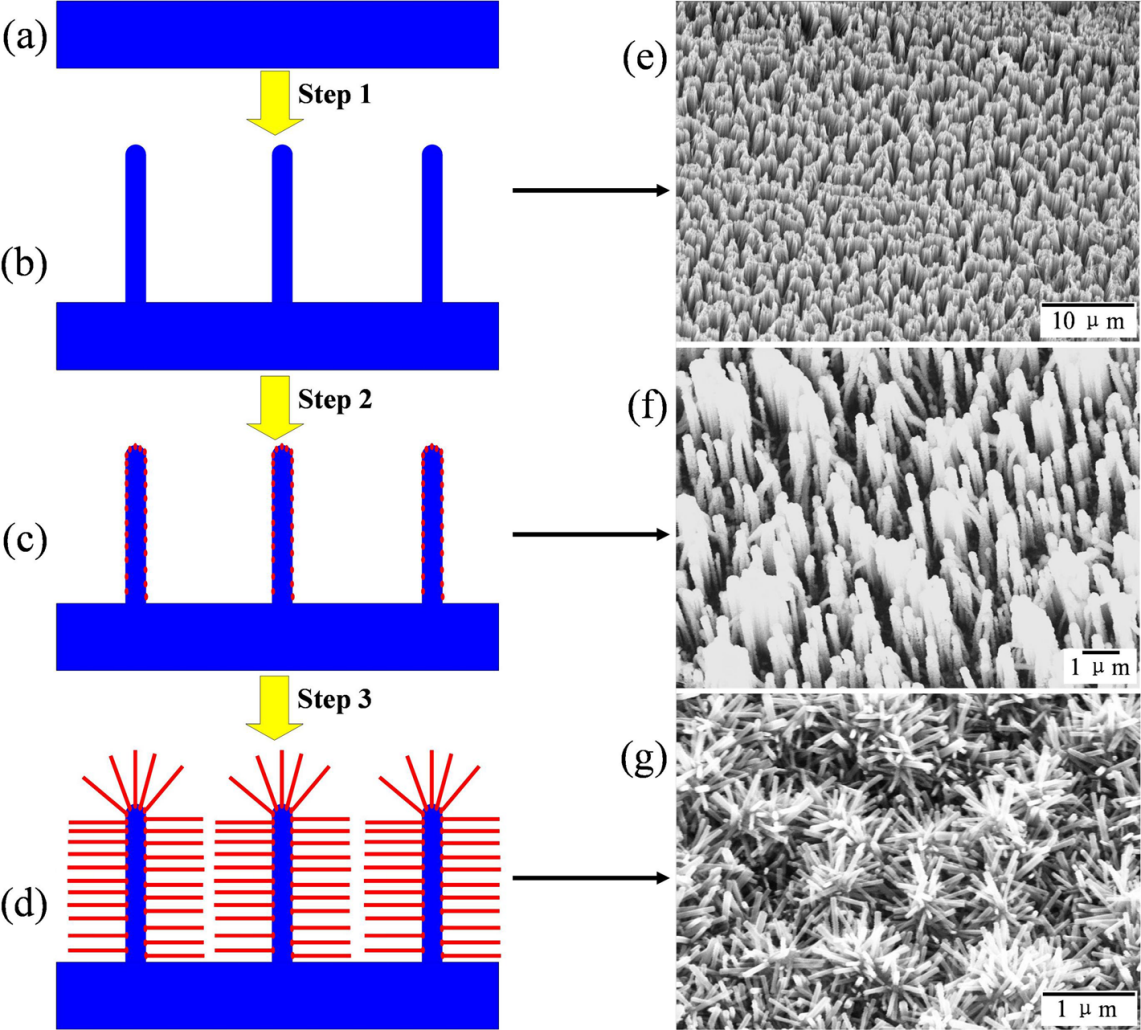
**Steps to synthesize branched ZnO/Si nanowire arrays (left panels) and corresponding SEM images (right panels).** The Si substrate **(a)**, the growth of Si nanowire arrays by chemical etching **(b)**, the deposition of ZnO thin film by magnetron sputtering as a seed layer on the Si nanowires surface **(c)**, the growth of ZnO nanowire arrays by hydrothermal method **(d)**, SEM images of the bare Si nanowire arrays **(e)**, the Si nanowire arrays decorated with ZnO nanoparticles **(f)**, and the branched ZnO/Si nanowire arrays with hierarchical structure **(g)**.

The synthesis procedure of branched ZnO/Si nanowire arrays with hierarchical structure in this study could be divided into three steps, as outlined by a schematic diagram in the left panels of Figure 1. First, crystalline Si nanowire arrays were prepared by wet chemical etching of Si substrates in a modified Piret's method [21]. In detail, the Si substrates were sequentially cleaned by ultrasonication in absolute toluene for 10 min, acetone for 10 min, ethanol for 10 min, and piranha solution (H_2_SO_4_ and H_2_O_2_ in a volume ratio of 3:1) at 80°C for 2 h, each of which was followed by copious rinsing with distilled water. After blow drying with nitrogen, the substrates were immediately immersed in aqueous solution of 5.25 M HF and 0.02 M AgNO_3_ in a Teflon vessel for a galvanic displacement reaction at room temperature. Post etching for a certain amount of time, the substrates were transferred to the solution of HCl/HNO_3_/H_2_O in a volume ratio of 1:1:1 overnight to remove the reduced Ag nanoparticles during the chemical etching. The substrates were then thoroughly rinsed with deionized water and dried in air.

Next, a layer of ZnO film with 25 nm in thickness was deposited on the surface of the Si nanowire arrays by a radio-frequency magnetron sputtering system. In order to achieve a uniform distribution of the seed layer, the sputtering was performed in a working pressure of 1.5 mTorr with a deposition rate of 3 nm/min. Afterward, the substrates were transferred into an oven and annealed at 500°C in nitrogen atmosphere for 30 min to obtain a tough adherence between the seed layer and the Si backbones.

Last, hierarchically branched ZnO nanowires were synthesized on the top and sidewall of the Si nanowires by a hydrothermal growth approach. In brief, the seeded samples were soaked vertically in aqueous solution of 25 mM Zn(CH_3_COO)_2_ · 2H_2_O and 25 mM C_6_H_12_N_4_ at 90°C in a glass beaker supported by a magnetic stirring apparatus. The hydrothermal process was conducted for a time period to control the length of the ZnO nanowires. After the reaction, the as-grown samples were removed from the solution, rinsed with deionized water, and then dried in air.

### Characterization

The morphology and size distribution of the products were characterized by a LEO-1530 field-emission SEM (Carl Zeiss AG, Oberkochen, Germany) with an accelerating voltage of 20.0 kV. Chemical composition of the specimens was analyzed using an EDS as attached on the SEM. Structural quality of the nanowire arrays was evaluated by an X’Pert PRO XRD (PANalytical Instruments, Almelo, Netherlands) with Cu K_α_ radiation (*λ* = 1.54056 Å). The PL spectra of the samples were collected on a Hitachi F-7000 fluorescence spectrophotometer (Hitachi, Tokyo, Japan) with an excitation wavelength of 325 nm. Optical reflectance measurements were performed on an Agilent Cary-5000 UV-vis-NIR spectrophotometer (Agilent Technologies, Sta. Clara, CA, USA). All the measurements were carried out at room temperature in normal conditions.

## Results and discussion

The structural evolution of the as-grown specimens that underwent 30-min chemical etching and 2-h hydrothermal growth (S30Z2) is presented in the right panels of Figure 1. It can be seen that after chemical etching in step 1 (Figure 1e), free-standing Si nanowire arrays in a wafer scale are produced on the substrate surface in a vertical alignment. The Si nanowire arrays have a length of about 2.5 μm and a diameter ranging between 30 and 150 nm. The growth rate of the nanowire length is about 1.4 nm/s and almost keeps constant for different durations. The structure, growth rate, and diameter of the Si nanowires are primarily restricted by the components and concentration of etching solution, as corroborated by the following experiments. A layer of ZnO nanoparticles is subsequently deposited on the Si nanowire array in step 2 (Figure 1f). Due to the isotropic characteristic of the sputtering system, the ZnO nanoparticles conformally coat on the nanowires and induce a rough sidewall surface. After hydrothermal growth in step 3 (Figure 1g), branched ZnO nanowires grow hierarchically on the surface of the Si nanowires, which fills up the space between the Si nanowires and presents a flower shape on each Si nanowire tip for the radial growth.

**Figure 2:**
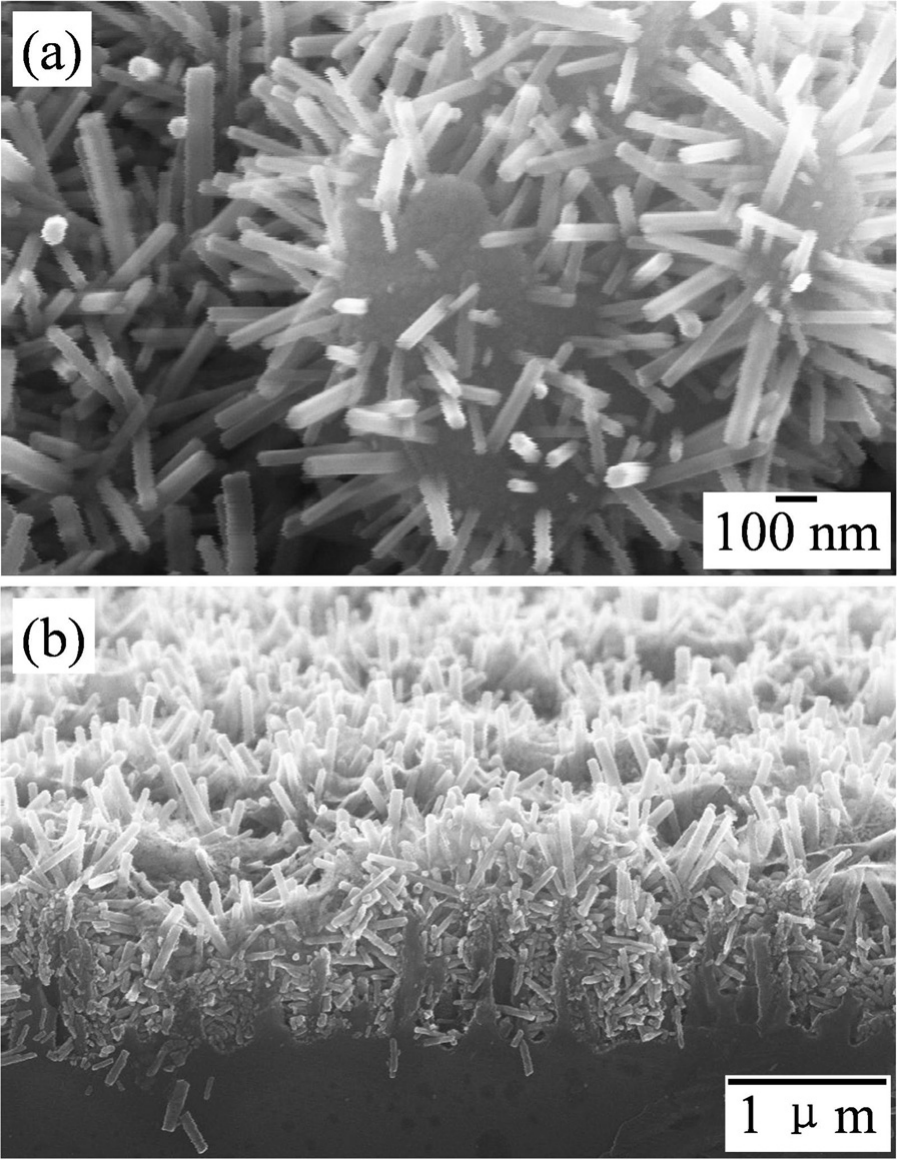
SEM images of branched ZnO/Si nanowire arrays: (a) magnified view and (b) cross-sectional view.

The heterogeneous nanowire structure is more obvious in the magnified and cross-sectional SEM images in Figure 2. The branched ZnO nanowires grow nearly in the normal direction to the Si nanowire surface. They have a hexagonal cross section and grow along the *c* axis of the wurtzite crystal. This is also confirmed by the following XRD pattern of the specimen. The distribution of ZnO nanowires seems non-uniform over the Si nanowire surface, which may be due to the non-uniformity of Si nanowire diameters from the chemical etching and the uneven coating of ZnO seed layer from sputtering. The mean diameter of ZnO nanowires is around 35 nm and is almost independent to the site of the Si nanowires. However, the length of ZnO nanowires is strongly dependent on the nanowires' location. It decreases from approximately 700 nm on top of the Si nanowires to approximately 80 nm in the bottom. As in the case of TiO_2_/Si nanostructure growth [22], the longer branches on top of the Si nanowires stem from the easy access of growth precursors with higher reactant concentration and less spatial hindrance from diffusion. It is found that the growth rate of the ZnO nanowires on top of the Si backbones is about 6 nm/min for the first 2.5 h and decreases drastically afterwards. Thus, the length of ZnO branches can be increased by prolonging the hydrothermal growth or repeating the growth in another fresh solution [23], and the length uniformity can be improved by growing ZnO nanowires on longer Si nanowires or on an array with larger spaces between the Si nanowires as created by combining latex mask and chemical etching [9].

**Figure 3:**
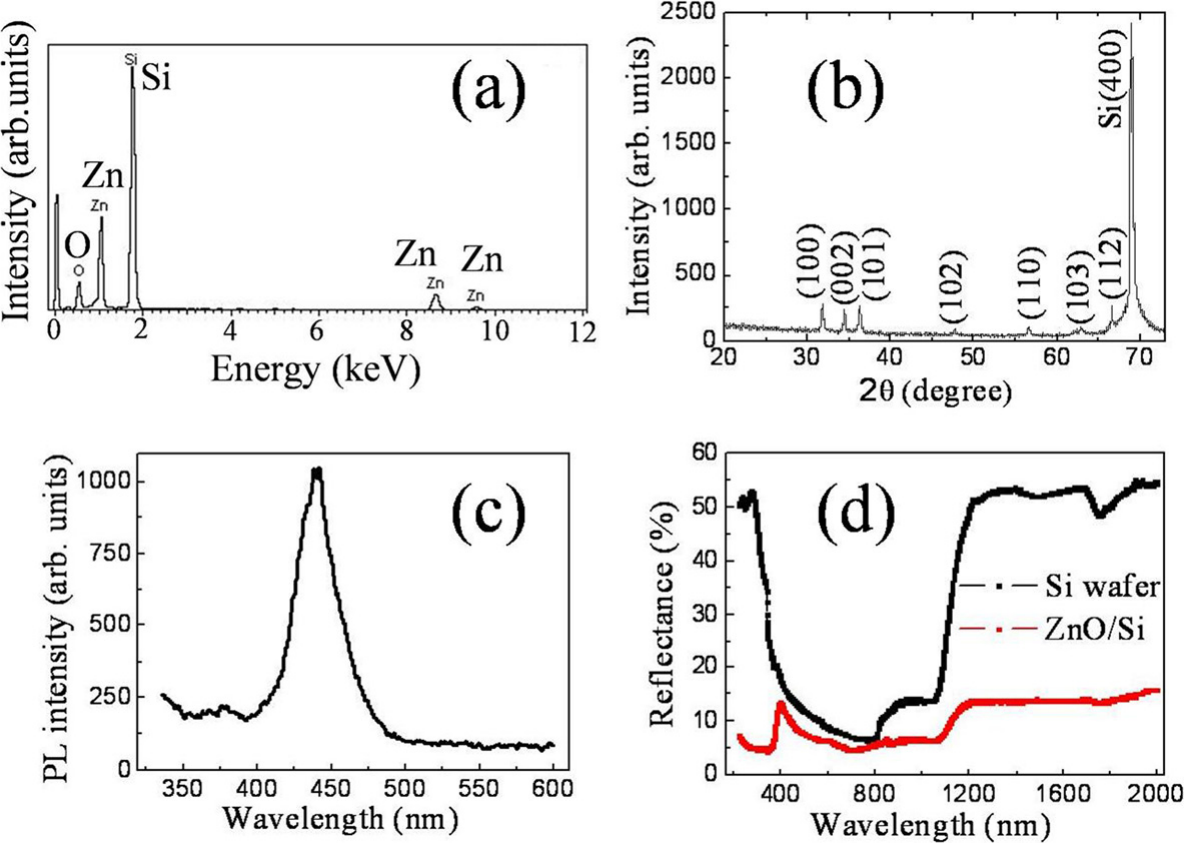
**Optical responses of branched ZnO/Si nanowire arrays. (a)** EDS spectrum. **(b)** XRD spectrum. **(c)** PL spectrum. **(d)** Reflectance. The reflectance of silicon wafer is also supplied in (d) for comparison.

Besides morphologic characterization, the final products were also systematically investigated by EDS, XRD, PL spectrum, and reflectance in order to elucidate the chemical composition, crystal structure, and optical properties. Figure 3a shows the EDS spectrum of the S30Z2 sample. Only signals originating from the elements of O, Zn, and Si are detected in it. Quantitative analysis yields a ratio of Si/Zn/O at about 3:1:1 (within a precision of 5%), thus, ensuring a stoichiometric ZnO composition in the branches of the hierarchical specimen. The excessive Si ratio possibly comes from the Si backbones that receive larger part of the detection electrons.

Figure 3b presents the XRD pattern of the S30Z2 specimen. Except a peak originating from the Si backbones and substrate, all the diffraction peaks are well indexed to those of hexagonal wurtzite ZnO (ICSD no. 086254), and no diffraction peaks of any other phases are detected. Moreover, there is no dominant peak in the wurtzite structure, which should be a result of the random orientation of the ZnO nanowires on the Si nanowire surface, as well supported by the SEM images in Figures 1g and 2.

The PL spectrum of the S30Z2 sample shown in Figure 3c consists of a weak ultraviolet peak at around 375 nm and a dominant blue emission at 440 nm with a broad feature in the range of 392 to 487 nm. The ultraviolet band corresponds to the near band-edge emission from ZnO branches [7,24], while the blue band is generally ascribed to the radial recombination of a photogenerated hole with electron in a single ionized oxygen vacancy in the surface lattice of the ZnO [25]. However, the visible emission may also be related to the surface defects within silicon oxide layer on the Si backbones, as the silicon surface is facile to be oxidized by the ambient oxygen and its emission band seats in the similar wavelength range [26]. Our experiments (not show here) indicate as well that only the blue emission band is present for the Si nanowire arrays, and the ultraviolet emission band is strengthened when the ZnO branches become denser or longer. Therefore, the weak ultraviolet emission and dominant blue band in the PL spectrum demonstrate the existence of ZnO and a large number of oxygen vacancies in the as-grown specimen.

A comparison of the hemispherical reflectance of the branched ZnO/Si nanowire arrays and a flat silicon wafer is provided in Figure 3d. The reflectance of the arrays is less than 15% over the wavelength range from ultraviolet to the mid-infrared region, which is drastically decreased relative to that of the silicon wafer. This significant property suggests that the nanotrees might be a promising candidate of antireflective surfaces or photoelectronics and photocatalysis for sunlight harvest. The ultralow reflectance of the specimen may result from the enhanced light-trapping and scattering for rough surface and large surface area of the nanotree arrays, multiple scattering of light within the hierarchical structure, as well as an effective refractive index (RI) gradient from air (RI ≈ 1.0) through ZnO nanowire array (RI ≈ 2.0) to Si nanowire array and substrate (RI ≈ 3.5) [18]. In addition, the abrupt drop in reflection is originated from band-edge absorption of the specimen [27]. The direct and indirect bandgaps of the components can thus be estimated by the onset points, which are 397 nm (equal to 3.123 eV) for the direct bandgap of ZnO nanowire branches and 1,221 nm (equal to 1.015 eV) for the indirect bandgap of Si nanowire backbones. In contrast to the Si wafer value 1,213 nm (equal to 1.022 eV) or to the general value of bulk materials, 3.37 eV for ZnO [7] and 1.12 eV for Si [5], the bandgaps of the as-grown specimen are found to be faintly narrowed down, suggesting ideal components of the object. The small difference may be due to the presence of ionic vacancies and structural defects in the nanotrees, as testified in the PL spectrum.

**Figure 4:**
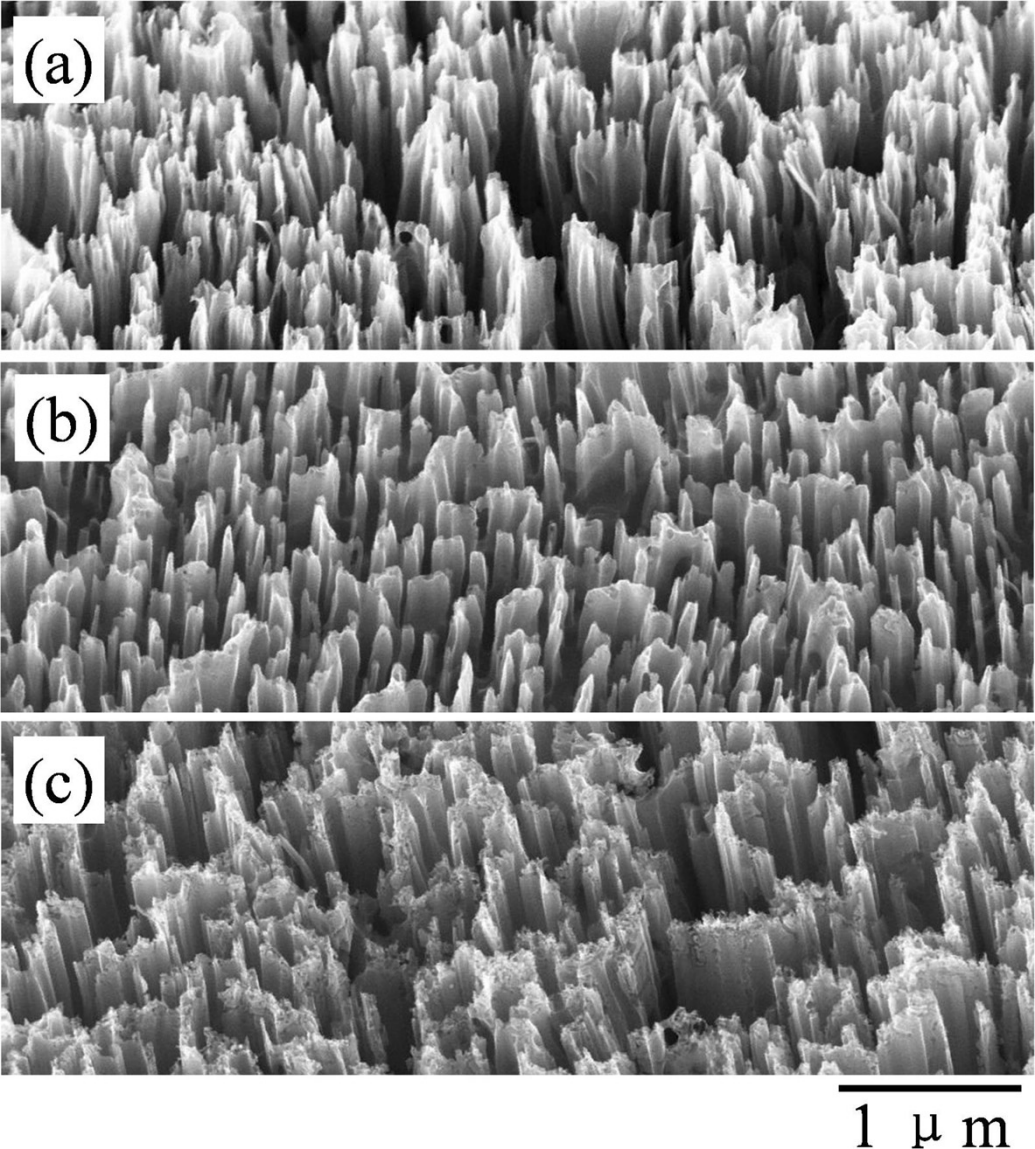
**SEM images of Si nanowire arrays prepared at room temperature in different solution. (a)** Substrate directly immersed in HF/AgNO_3_ (5.25/0.02 M) aqueous solution for 20 min. **(b)** Substrate immersed in HF/AgNO_3_ (4.6/0.01 M) aqueous solution for 60 s and subsequently transferred into HF/Fe(NO_3_)_3_ (4.6/0.135 M) aqueous solution for 20 min. **(c)** Substrate immersed in HF/AgNO_3_ (4.8/0.01 M) aqueous solution for 10 s and subsequently transferred into HF/H_2_O_2_ (4.6/0.4 M) aqueous solution for 15 min.

The above results and analysis confirm that branched ZnO/Si nanowire arrays with hierarchical structure can be facilely grown on the silicon substrate in a wafer scale by the cost-effective methods. However, as the procedure includes chemical etching for the silicon backbones and hydrothermal growth of the ZnO branches, different synthesis parameters may cause serious influences on the structure and performance of the ZnO/Si nanowire arrays. For this reason, we systematically study crucial influences of the key parameters on the structure of the objects. First, the influence of etching solution on the silicon backbones is investigated, and the results are shown in Figure 4. We can see in Figure 4a that the Si nanobelt or nanowire arrays orient vertically on the Si substrate when the substrate was immersed into aqueous solution of HF/AgNO_3_ (5.25/0.02 M) at room temperature for 20 min. The arrays exhibit a rough surface for irregular tapered tip ends of the nanostructure. In contrast, when the substrate was first immersed in aqueous solution of HF/AgNO_3_ (4.6/0.01 M) for 60 s and subsequently transferred into aqueous solution of HF/Fe(NO_3_)_3_ (4.6/0.135 M) for 20 min (see Figure 4b), the rough surface disappears and the vertically aligned Si nanowire arrays with smooth sidewall surface present in a better order. Nevertheless, when the substrate was changed to be immersed in aqueous solution of HF/AgNO_3_ (4.8/0.01 M) for 10 s and subsequently transferred into aqueous solution of HF/H_2_O_2_ (4.6/0.4 M) for 15 min (see Figure 4c), slanted nanowire arrays with porous tip ends arise on the Si substrate instead of vertically aligned nanostructure. In the growth procedure, the formation of one-dimensional silicon nanostructures is based on electroless silver deposition on silicon and silver-nanoparticle-catalyzed chemical etching of silicon in HF-based solution [28]. As the difference among the three methods is introducing an oxidant of Fe(NO_3_)_3_ or H_2_O_2_ in the etchant solution, it is reasonable to believe that the different morphologies of the silicon nanostructures originate from redox potential of the oxidants. Namely, the Fe^3+^/Fe^2+^ system has a lower positive redox potential than that of Ag^+^/Ag couple [28], which reduces the etching speed of the silicon substrate in contrast to the former solution and promotes the morphology of the product. But for O^1−^/O^2−^ system, the positive redox potential is much higher than that of Ag^+^/Ag couple [29], which enhances the etching ability of the solution. Owing to the fast etching of the substrate, some Ag particles may reside on the nanowire top surface randomly and metal-assisted chemical etching continues locally, which induces the tapered tip ends in Figure 4a and porous tip ends in Figure 4c. The tapered and porous tip ends tend to be penetrated by the following ZnO seed layer deposition. Based on the above analysis, we can conclude that a moderate etching speed is crucial for achieving a well-aligned nanowire array with solid and round surface. In fact, the morphology and structure of the Si nanowire arrays can also be tailored by other parameters, such as etching period [28], solution concentration [29] and temperature [30], crystalline character of the substrates [30,31], as well as surface treatment [32]. These are beyond the scope of this article and can be found in references and relative researches.

**Figure 5:**
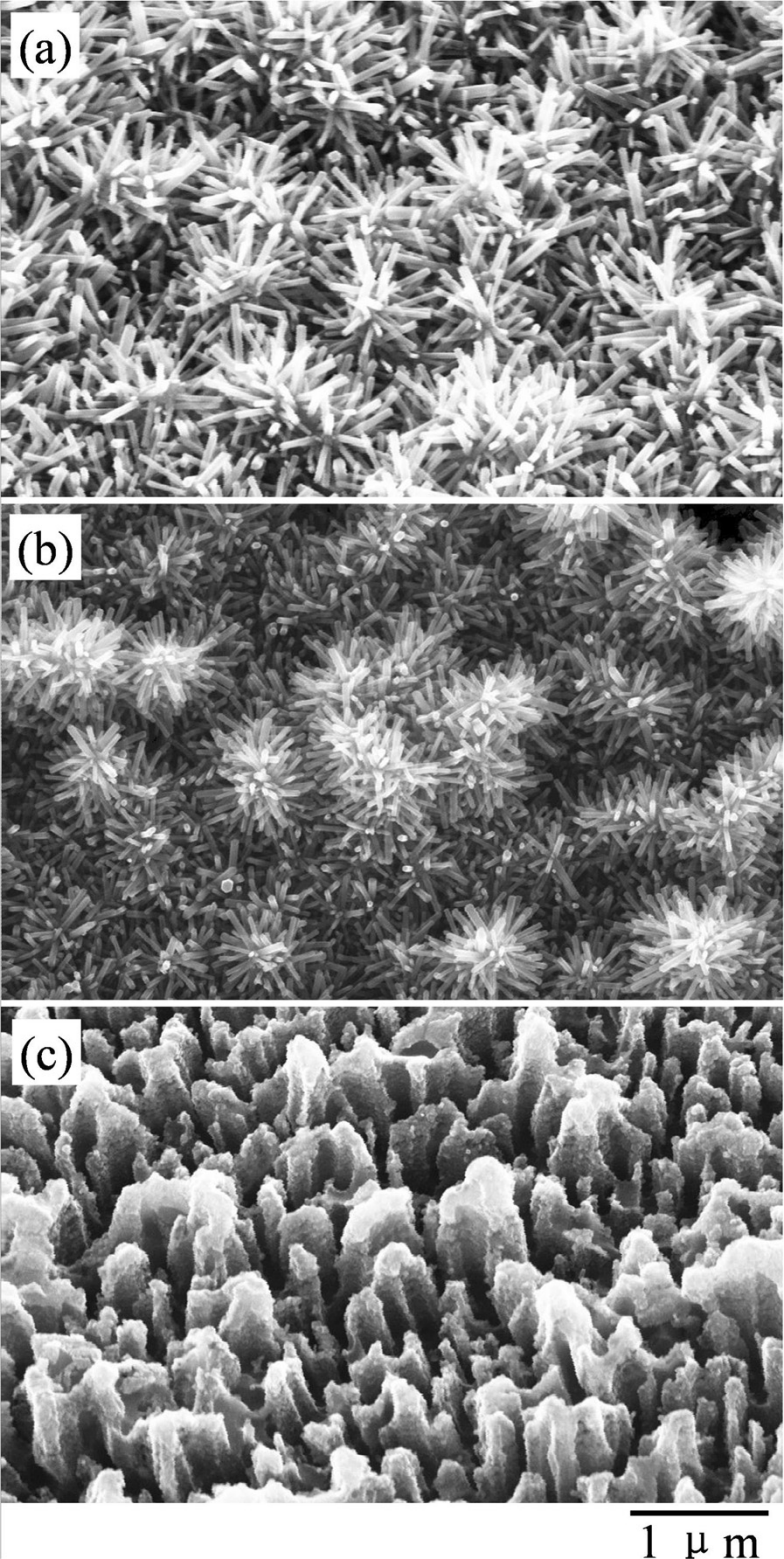
**SEM images of products prepared in different substrate directions in solution: (a) vertical, (b) facedown, and (c) faceup.** The Si nanowire arrays were capped with ZnO seed layer before hydrothermal growth.

Figure 5 compares SEM images of the products prepared in a similar procedure of S30Z2 sample but with different substrate directions relative to the solution surface during the hydrothermal growth. For the substrate immersed vertically into the precursor solution, branched ZnO nanowires with wurtzite crystal structure grow radially and form a flower shape on each of the Si backbones. The morphology of the product prepared by immersing the substrate facedown into the reaction solution is the same as that of the former case, and both seem to possess an identical growth speed as the length of ZnO nanowires is similar. Nevertheless, for the third case with a faceup direction, the ZnO nanowire arrays disappear on the Si backbones. The Si nanowires tend to bundle up and their surface becomes much rougher in contrast to the Si nanowires with seed layer in Figure 1f. It is well known that water molecules run violently at high temperature, which may cause deformation of adjacent nanowire tips into clusters for reducing the total energy. Meanwhile, the condensation of the ZnO nanoparticles from the growth solution results in the rough surface of the Si nanowires. The observation indicates that the presence of gravity gradient is a key issue for the growth of ZnO nanowire arrays. Otherwise, only the condensation of the ZnO nanoparticles takes place in a form of film on the seed layer. The intrinsic mechanism possibly lies in the specific character of chemical reactions in the aqueous solution as well as the thermodynamics and kinetics of ZnO growth, which is under further exploration.

**Figure 6:**
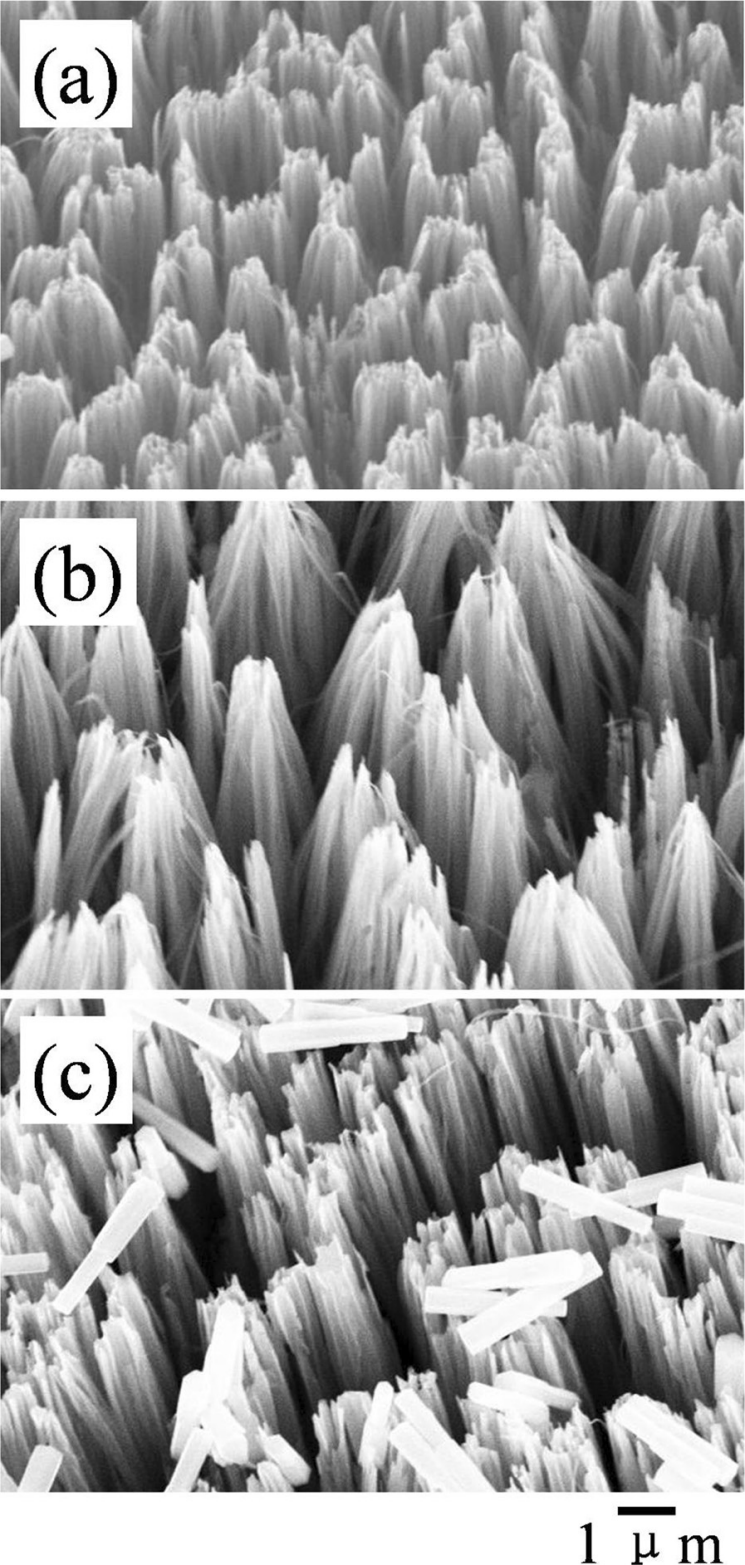
**SEM images of products prepared in different substrate directions in solution on the Si nanowire arrays: (a) vertical, (b) facedown, and (c) faceup.** The Si nanowire arrays were not capped with ZnO seed layer before hydrothermal growth.

It is worthwhile to point out that the seed layer is another important factor in the growth of branched ZnO nanowires. Figure 6 shows the SEM images of the products prepared by 30-min etching and 2-h hydrothermal growth but without the seed layer deposition. The substrates were also soaked in different directions relative to the solution surface during the hydrothermal growth. It is found that after hydrothermal growth, all the Si nanowire arrays exhibit original morphologies except the bending of the nanowires to form sheaf-like structures in some specimens. The ZnO nanowires or nanorods are also created but disperse randomly on the Si nanowire arrays surface and are removed easily by subsequent cleaning. The sheaf-like structures in Figure 6 are due to the surface tension force presence in the high-temperature solution as well as in the drying process that deforms adjacent nanowire tips into clusters. For the disappearance of ZnO nanowire branches, it is well known that the crystal structure and chemical bonds of ZnO substance are different from those of Si substance. The precursors in the aqueous solution do not automatically nucleate on the Si nanowire and grow to be a ZnO nanowire during the hydrothermal process. The appearance of ZnO nanowires or nanorods in the solution after the hydrothermal growth may stem from the impurities acting as nucleation sites since the reagents in the experiment are not of ultra-purity. In this regard, the seed layer on the Si nanowire surface plays an important role in the growth of branched ZnO/Si nanowire arrays as it provides nucleation sites and determines the growing direction and density of the ZnO nanowire arrays for reducing the thermodynamic barrier.

## Conclusions

Branched ZnO/Si nanowire arrays with hierarchical structure were synthesized by a three-step process, including the growth of crystalline Si nanowire arrays as backbones by chemical etching of Si substrates, the deposition of ZnO thin film as a seed layer by magnetron sputtering, and the fabrication of ZnO nanowires arrays as branches by hydrothermal growth. During the synthesis procedure, an etchant solution with an appropriate redox potential of the oxidant was vital for a moderate etching speed to achieve a well-aligned Si nanowire array with solid and round surface. Meanwhile, the presence of gravity gradient was a key issue for the growth of branched ZnO nanowire arrays. The substrate should be placed vertically or facedown in contrast to the solution surface during the hydrothermal grown. Otherwise, only the condensation of the ZnO nanoparticles took place in a form of film on the substrate surface. The seed layer played another important role in the growth of ZnO nanowire arrays, as it provided nucleation sites and determined the growing direction and density of the nanowire arrays for reducing the thermodynamic barrier.

## Competing interests

The authors declare that they have no competing interests.

## Authors' contributions

SH designed and performed the experiments, analyzed the data, and drafted the manuscript. QY helped prepare and characterize the samples and analyze the data. BY, DL, and RZ participated in the preparation of the samples. SL and JK participated in the final data analysis and critical review of the manuscript. All authors read and approved the final manuscript.
